# The use of personal protection equipment does not negatively affect paramedics’ attention and dexterity: a prospective triple-cross over randomized controlled non-inferiority trial

**DOI:** 10.1186/s13049-021-00990-3

**Published:** 2022-01-10

**Authors:** Calvin Lukas Kienbacher, Jürgen Grafeneder, Katharina Tscherny, Mario Krammel, Verena Fuhrmann, Maximilian Niederer, Sabine Neudorfsky, Klaus Herbich, Wolfgang Schreiber, Harald Herkner, Dominik Roth

**Affiliations:** 1 Department of Emergency Medicine, Medical University of Vienna, Währinger Gürtel 18-20, 1090 Vienna, Austria; 2 Department of Clinical Pharmacology, Medical University of Vienna, Währinger Gürtel 18-20, 1090 Vienna, Austria; 3 Emergency Medical Services Vienna, Radetzkystraße 1, 1030 Vienna, Austria; 4 PULS – Austrian Cardiac Arrest Awareness Association, Lichtentaler Gasse 4/1/R03, 1090 Vienna, Austria

**Keywords:** Cardiopulmonary resuscitation, Personal protection equipment, Concentration, Neurocognition, COVID-19

## Abstract

**Background:**

The COVID-19 pandemic led to widespread use of personal protection equipment (PPE), including filtering face piece (FFP) masks, throughout the world. PPE. Previous studies indicate that PPE impairs neurocognitive performance in healthcare workers. Concerns for personnel safety have led to special recommendations regarding basic life support (BLS) in patients with a potential SARS-CoV-2 infection, including the use of PPE. Established instruments are available to assess attention and dexterity in BLS settings, respectively. We aimed to evaluate the influence of PPE with different types of FFP masks on these two neuropsychological components of EMS personnel during BLS.

**Methods:**

This was a randomized controlled non-inferiority triple-crossover study. Teams of paramedics completed three 12-min long BLS scenarios on a manikin after having climbed three flights of stairs with equipment, each in three experimental conditions: (a) without pandemic PPE, (b) with PPE including a FFP2 mask with an expiration valve and (c) with PPE including an FFP2 mask without an expiration valve. The teams and intervention sequences were randomized. We measured the shift in concentration performance using the d2 test and dexterity using the nine-hole peg test (NHPT). We compared results between the three conditions. For the primary outcome, the non-inferiority margin was set at 20 points.

**Results:**

Forty-eight paramedics participated. Concentration performance was significantly better after each scenario, with no differences noted between groups: d2 shift control versus with valve − 8.3 (95% CI − 19.4 to 2.7) points; control versus without valve − 8.5 (− 19.7 to 2.7) points; with valve versus without valve 0.1 (− 11.1 to 11.3) points. Similar results were found for the NHPT: + 0.3 (− 0.7 to 1.4), − 0.4 (− 1.4 to 0.7), 0.7 (− 0.4 to 1.8) s respectively.

**Conclusion:**

Attention increases when performing BLS. Attention and dexterity are not inferior when wearing PPE, including FFP2 masks. PPE should be used on a low-threshold basis.

## Background

The current COVID-19 pandemic led to widespread use of personal protection equipment (PPE) on a global level [1–3]. Prior studies indicate that masks and gowning may impair fine motor skills and the ability to concentrate. This may not only be the case during exhaustive physical work but also whilst performing routine processes [4–7]. Various reasons for this have been considered, including increased humidity underneath the mask, rebreathing of carbon dioxide and increased breathing resistance [7–10]. Other important points might be the impairment of vision and “soft skills” like communication [4]. Recently, we showed that the use of COVID-19 PPE does not impair the quality of cardiopulmonary resuscitation itself [11]. The impact of such equipment on the neurocognitive performance of healthcare providers, however, remains unclear.

Performing high-quality basic life support (BLS) leads to physical and mental stress, even in healthcare workers including physicians [12–14]. Its complexity substantially increases with the amount of tasks performed simultaneously [15]. This may impair the quality of care. Performing BLS in the setting of emergency medical service requires multitasking skills of ambulance professionals. Providers have to assess the patient’s condition and plan further treatment while simultaneously performing cardiopulmonary resuscitation (CPR), consisting of chest compressions and ventilation. The European Resuscitation Council (ERC) guidelines recommend 30 chest compressions at a frequency of 100 to 120 per minute followed by 2 rescue breaths in an alternating manner [16]. The BLS providers’ capacity to concentrate is paramount to achieve good clinical patient outcomes. Aside from these aspects, operating an EMS vehicle with decreased attention capacity poses a threat to the public environment [17].

Special recommendations regarding BLS during the COVID pandemic are available. These include the use of protective gowning with masks, gloves, and eye shields to protect medical personnel [18].

Different types of filtering face piece (FFP) masks are frequently used by medical professionals: Some masks have expiration valves, others do not. The valve opens on expiration and makes it easier to exhale. The former models protect only the wearer from infectious diseases, the latter also serve to protect others. The number following the abbreviation “FFP” indicates the level of protection. FFP2 and FFP3 masks have frequently been used in many countries during the COVID 19-pandemic [19].

Physical and mental strain are issues of special importance in the field of prehospital emergency medicine, a field where multitasking is an important competence and resources are often sparse. Attention is considered a cornerstone of neurocognitive performance and of paramount importance for all tasks of daily life [20]. Attention can be evaluated using the d2-test. This test was developed in the early 1960s to evaluate drivers’ proficiency in Germany. It has been validated extensively and is considered to be highly reliable [21, 22]. Within this cancellation test subjects have to search for patterns within a sequence of letters. Only “d”s with a sum of two lines above and/or below them are to be marked. Several distractors are included (e.g. “d”s with only one line or “p”s with one or two lines). Due to the simple and convenient nature of the procedure it is used widely, e.g., in clinical psychology, medicine and research [23–25].

The nine-hole peg test can be used to evaluate dexterity. Subjects have to remove nine small pins from holes in a board and put them back as fast as possible using the dominant hand. Time is measured and greater speed indicates better performance [26]. This test has been used in the field of medicine, including multiple previous studies to test dexterity as a measure of strain after performing CPR in settings similar to ours [27–31].

To the best of our knowledge, no previous study has yet investigated the influence of PPE on the neurocognitive performance of healthcare workers during and after high-performance critical care procedures such as CPR.

## Methods

This study was a prospective triple-cross over randomized controlled non-inferiority trial.

### Setting and intervention

We described the study-scenario in detail in a previous publication, where we investigated the effect of PPE on the quality of CPR but didn’t evaluate the healthcare workers’ perspective [11]. This part of our trial focusses on the strain on the mental reserves of ambulance crews. We block-randomized emergency medical technicians (EMTs) to teams of two. All teams performed standardized BLS scenarios. This included walking quickly up and down three flights of stairs while carrying standard equipment (backpack and oxygen cylinder). Following this, they performed 12 min of BLS on a manikin (qCPR ResusciAnn, Laerdal^®^, Norway) in accordance with current ERC guidelines. Every 2 min the team-members swapped between executing chest compressions and performing ventilation. Participants adhered to a rest period of 30 min between each scenario.

For the control scenario, participants wore their standard PPE consisting of EMS uniform, safety boots and examination gloves. For the intervention scenarios, an overall, goggles and either of the following two masks was added: an FFP2 mask with an expiration valve (“Meditrade Respima^®^ EEC” (Meditrade^®^ GmbH, Germany)), or an FFP2 mask without an expiration valve (“Yao Wang Medical Protective Face Mask” (Qingzhou Yaowang Pharmaceuticals Co. Ltd., China)). The Meditrade masks were certified to the N95-standard, whereas the Yao Wang masks were certified to the KN95-standard. These standards a virtually identical for the purpose of this paper in regard to flow rate, maximum inhalation and exhalation resistance. Scenarios were performed in a randomized order.

### Measurement

We tested concentration performance using the d2 test battery including its subsets before and after each scenario [32].

As a secondary outcome, we investigated dexterity as a measure of psychomotoric strain using the nine-hole peg test (NHPT).

A psychologist experienced in clinical testing (JG) performed both tests.

### Statistical analysis

The primary outcome was the shift in overall concentration performance after each scenario, as measured by the d2 test.

We tabulated all results for each of the three scenarios and calculated absolute differences (with 95% confidence intervals) between the scenarios.

To assess potential carry over, we also analyzed the influence of the sequence in which participants completed the scenarios on the outcome, using regression modelling.

Secondary outcomes included the shift in dexterity, measured by the NHPT, as well as the d2-subscores, i.e., percentage of errors, errors of commission, errors of omission, and processed target objects. Analysis of the secondary outcomes followed the primary outcome. An individual provider served as the unit of analysis.

Sample size considerations were based on the non-inferiority of the primary outcome of shift in overall concentration performance. The reference values for good performance in the d2-test range from 184 to 207 points for 20- to 39-year-old individuals [32]. Based on previous experience, and the known distribution of d2-test results, a clinically relevant non-inferiority limit was defined as 20 points. Considering the reference range, drops or increases of twenty points indicate inferior (below the 25th percentile) or superior (above the 75th percentile) performance, respectively. We needed to include 40 participants to achieve a power of 0.8 at a significance level of 0.05. This was well within the 48 individuals needed to study quality of CPR as outlined in our previous paper [11].

MS Excel (Microsoft Corporation) and STATA 13SE (Stata Corporation) were used for data management and analysis. We conducted our study following the principles of the Declaration of Helsinki. All participants provided written informed consent before inclusion. The local ethics committee approved the study (#1520/2020).

### Patient and public involvement

Although improving the quality of CPR and post-resuscitation care for patients is the ultimate goal of this investigation, caregivers and their neurocognitive performance are the immediate study suspects. Involvement efforts therefore focused on those individuals, and patients or patient representatives were not involved.

We developed the research question and the study protocol together with representatives from the prehospital emergency provider community. EMS recruited the participants. After conclusion of the study, we performed non-structured interviews with all participants to get their feedback on the burden of participation and possible future improvements. Participants were debriefed with their provisional results as feedback.

## Results

We randomized forty-eight emergency medical service (EMS) providers into 24 teams of two (Table 1). There was no difference in baseline vital signs, as has previously been published [11].

**Table 1:** Demographic data of study participants

	Overall (N = 48)
Age, years (SD)	28 (8)
Female, n (%)	4 (8)
EMT qualification, n (%)	
Basic life support	20 (42)
Intermediate level	11 (23)
Advanced life support	17 (35)

*EMT* emergency medical technician, *SD* standard deviation

**Fig. 1:**
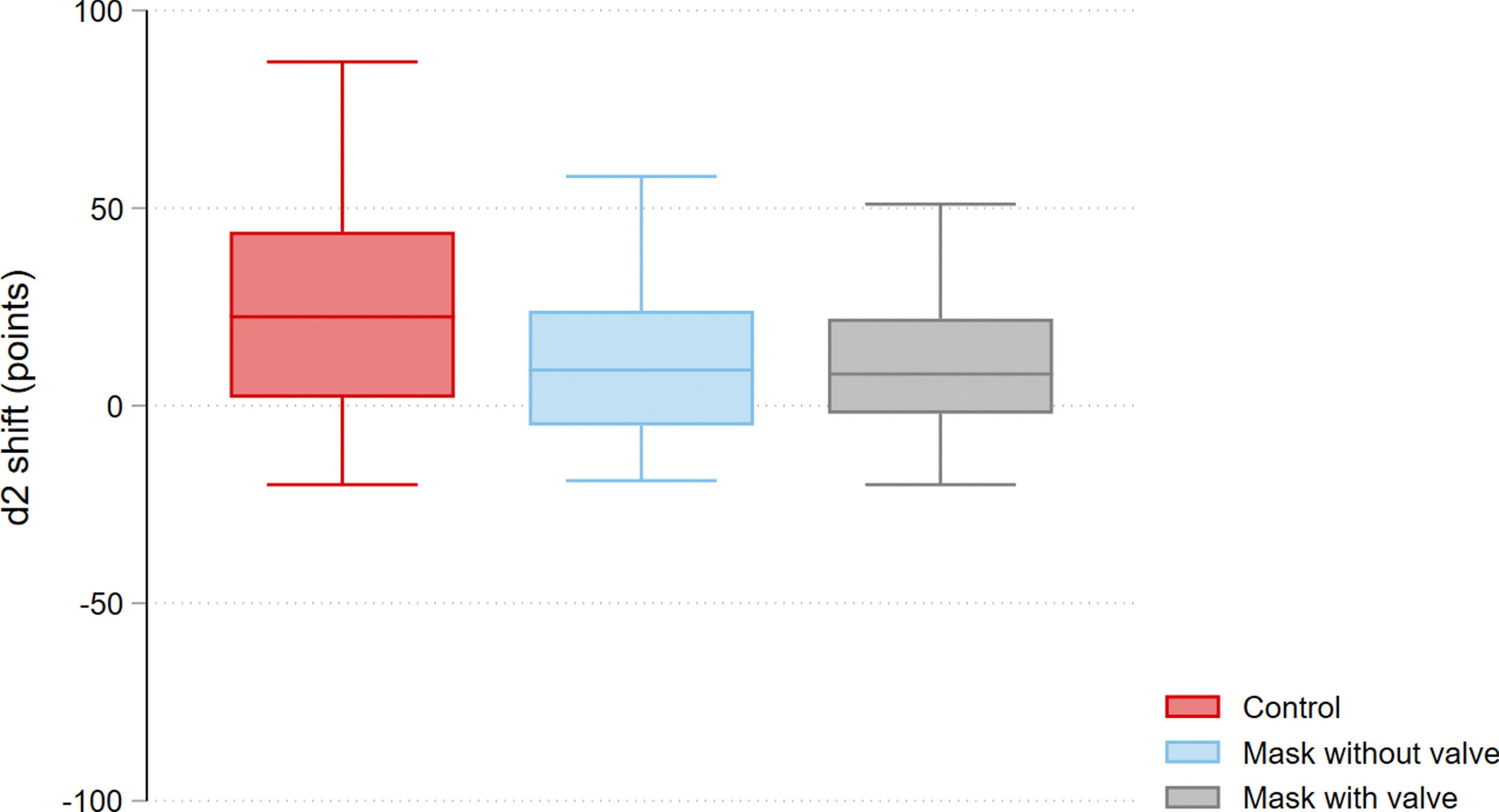
Shift of concentration performance as measured by the d2 test, per scenario

Concentration performance was significantly improved after performance of each of the scenarios, reflected in a positive d2 score shift: control + 20.2 (95% CI 9.3 to 31.7) points; with valve + 11.9 (95% CI 5.4 to 18.3) points; without valve + 11.7 (95% CI 6.2 to 17.2) points (see Fig. 1). There was no difference between groups (control vs. with valve − 8.3 (95% CI − 19.4 to 2.7) points, control vs. without valve − 8.5 (95% CI − 19.7 to 2.7) points, with valve vs. without valve 0.1 (95% CI − 11.1 to 11.3) points), indicating non-inferiority of one type of PPE over the other. Results of the d2-subscores were similar to those for overall concentration performance.

**Fig. 2:**
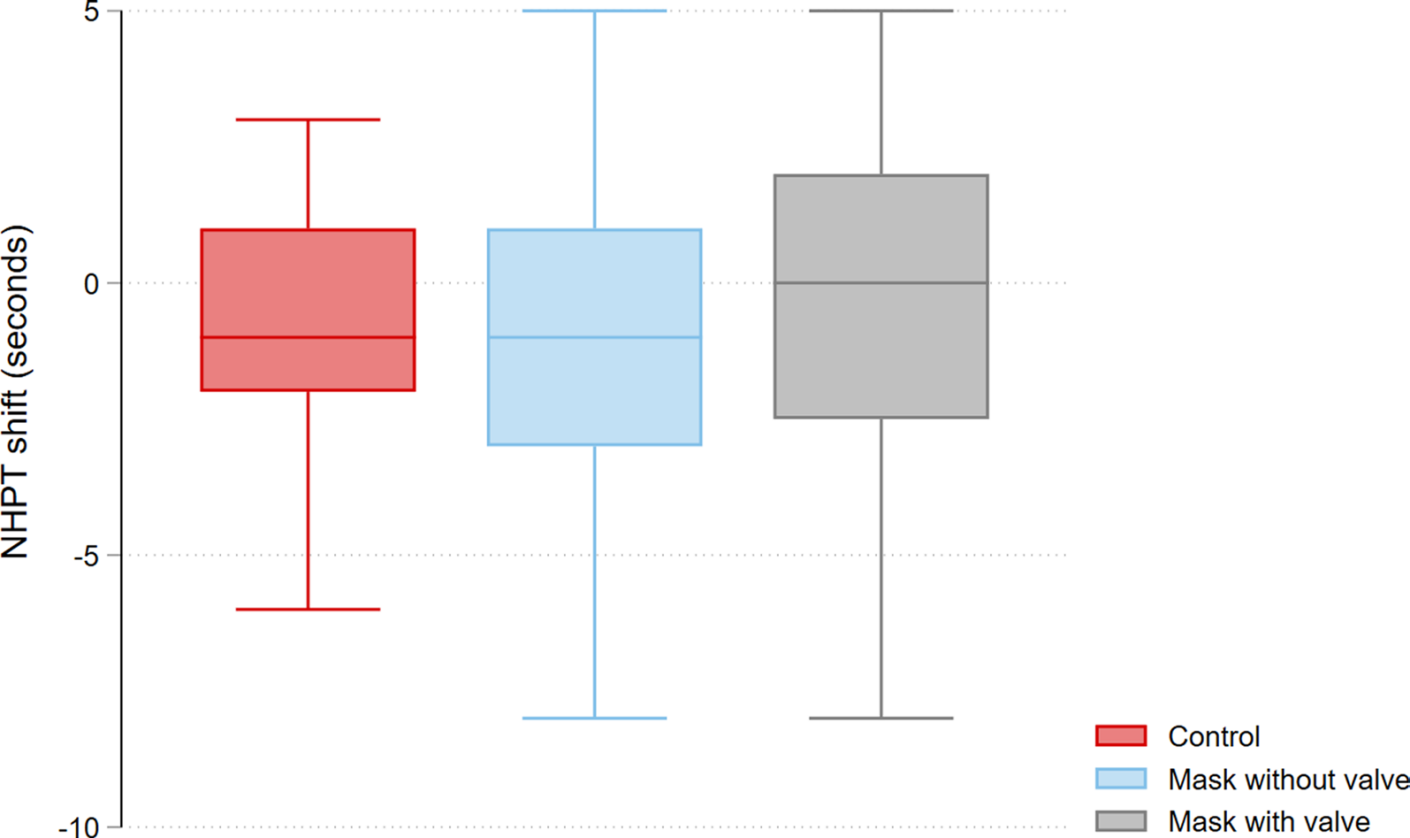
Shift of dexterity as measured by the nine-hole peg test (NHPT), per scenario

For the NHPT, there was no shift or minimal improvement: control − 0.6 (95% CI − 1.4 to 0.1) s, with valve − 0.3 (95% CI − 1.1 to 0.5) s, without valve − 1 (95% CI − 1.8 to − 0.3) s (see Fig. 2). As for the primary outcome, there was no significant difference in those shifts between groups (control vs. with valve + 0.3 (95% CI − 0.7 to + 1.4) s, control vs. without valve − 0.4 (95% CI − 1.4 to + 0.7) s, with valve vs. without valve + 0.7 (95% CI − 0.4 to + 1.8) s).

Table 2 provides a detailed summary of the findings.

**Table 2:** Primary and secondary outcomes

	Control	Mask without valve	Mask with valve
Concentration performance d2 (points)			
d2 shift (SD)	20.2 (37.5)	11.7 (18.6)	11.9 (22.1)
d2 shift, absolute difference versus control [95% CI]	–	− 8.5 [− 19.7, 2.7]	− 8.3 [− 19.4, 2.7]
d2 before BLS (SD)	181.4 (52.7)	189.9 (7.4)	191.1 (50.9)
d2 after BLS (SD)	201.5 (47.4)	201.6 (49.8)	203 (48.8)
d2 subscores			
Error rate (%)			
Subscore shift (SD)	− 0.1 (2.2)	0.2 (1.1)	− 0.1 (1)
Subscore shift, absolute difference versus control [95% CI]	–	0.3 [− 0.4, 0.9]	0 [− 0.6, 0.6]
Subscore before scenario (SD)	2 (7.6)	2.1 (10.2)	2.3 (9.5)
Subscore after scenario (SD)	1.9 (8.7)	2.3 (9.7)	2.2 (10)
Errors of commission (points)			
Subscore shift (SD)	0.2 (5.2)	0.6 (2.1)	− 0.2 (2)
Subscore shift, absolute difference versus control [95% CI]	–	0.4 [− 1, 1.8]	− 0.4 [− 1.8, 1]
Subscore before scenario (SD)	3.7 (13.1)	4 (19.1)	4.7 (19)
Subscore after scenario (SD)	3.9 (16.6)	4.6 (18.6)	4.5 (19.4)
Errors of omission (points)			
Subscore shift (SD)	− 6.6 (10.8)	− 2.5 (7.4)	− 1.8 (9.6)
Subscore shift, absolute difference versus control [95% CI]	–	4.1 [0.3, 7.9]	4.8 [1, 8.6]
Subscore before scenario (SD)	20 (25.2)	17 (22.1)	16.7 (21)
Subscore after scenario (SD)	13.4 (18.4)	14.5 (18.7)	14.9 (18.6)
Processed target objects (points)			
Subscore shift (SD)	13.8 (35.1)	9.8 (17.5)	9.9 (18.7)
Subscore shift, absolute difference versus control [95% CI]	–	− 4 [− 14.3, 6.3]	− 3.9 [− 14.1, 6.2]
Subscore before scenario (SD)	205 (46)	210.9 (44.5)	212.5 (43.7)
Subscore after scenario (SD)	218.8 (41.3)	220.7 (6.3)	222.4 (41.2)
Dexterity NHPT (s)			
NHPT shift (SD)	− 0.6 (2.5)	− 1 (2.6)	− 0.3 (2.8)
NHPT shift, absolute difference versus control [95% CI]	–	− 0.4 [− 1.4, 0.7]	0.3 [− 0.7, 1.4]
NHPT before scenario (SD)	24.8 (3.6)	24.2 (3.1)	24 (3.5)
NHPT after scenario (SD)	24.2 (3.1)	23.2 (2.8)	23.7 (3.1)

*CI* confidence interval, *NHPT* nine-hole peg test, *SD* standard deviation

Two subjects from the same team did not perform d2 testing before and after BLS in the scenario with a mask without a valve. There were no missing observations regarding the NHPT and no loss to follow up.

To test for training or carry-over effects, we analyzed the influence of the sequence in which participants performed scenarios on the outcomes. For the first run, there was a positive shift in concentration performance from before to after by 36.7 (SD 17.7) points, whereas for the second run this shift decreased to 5.7 (SD 15.9) points, and for the third run to only 0.9 (SD 31.4) points. The difference between first and second (− 31.0 points; 95% CI − 40.1 to − 21.8), and first and third run (− 35.8 points; 95% CI − 45 to − 26.6) were significant, with no differences between the latter two (− 4.8 points; 95% CI − 14.1 to 4.4). We found no influence of sequence on NHPT results.

## Discussion

Our data indicate that the use of various PPE does not negatively influence the neurocognitive performance of healthcare providers before and after performing BLS. This implies that the planning and execution of care for a patient suffering from cardiac arrest while wearing an FFP2 mask is possible without substantial impairment. In the prehospital setting, this is especially important and helpful with regards to operational concerns, including the patient’s transport. The ability to drive an ambulance vehicle with attention levels above average is an important safety factor in this setting.

Our findings differ from those of other trials. One study investigated the impact of PPE on the performance of surgeons and found their skills to be significantly impaired [4]. The task of performing BLS is different to performing surgery in an operating theater. This includes the kneeling position whilst performing CPR versus standing or sitting during an operation. In addition, CPR does not necessitate paying attention to maintaining a sterile environment. Another trial focused on the impact of a chemical, biological, radiological, and nuclear (CBRN) PPE on routine activities in respects to vascular access and airway management. Study subjects needed more time and more attempts to complete their tasks successfully [5].

A study by Loibner et al. found results similar to ours. The authors were able to show that PPE is generally well tolerated, although their gowning and setting of laboratory work differed substantially from the conditions found in our protocol [33].

The results of our study shed light on a huge blind spot. To our knowledge, no data on the cognitive performance of medical personnel who conduct the CPR exists. In a recently published literature review, Sedlár [34] found an increased interest in cognitive skills of EMS crew members. Yet most research was qualitative and none of the publications investigated a CPR scenario. To our knowledge, this is the first quantitative, experimental study investigating neurocognitive skills in this field. We hope that our study will foster interest and further research on this important topic.

We are aware of the fact that our study has some limitations. Firstly, we used a simulation setting. We aimed to create a scenario as close to reality as possible and imposed physical stress upon the participants before each scenario. Nevertheless, a simulation is unlikely to create exactly the same level of psychological pressure as a real situation.

Furthermore, one might argue that we assessed only a limited spectrum of neurocognitive performance, using just two tests. However, attention is generally considered to be the most important requirement to successfully complete both everyday and complex tasks [20]. More investigations in the same period of time would have been hardly feasible due to the nature of our experimental setup, i.e., rapid evaluation as soon as possible after the runs to minimize confounding. Extensive testing can cause additional mental strain and might therewith influence performance.

The d2 test and NHPT are thoroughly validated well-established tests for our research question [26, 35]. As described in the literature, we found a training effect for the d2 test. While there was a relevant difference between the before and after test for the first run (irrespective of which scenario participants were randomized to for their first run), this difference diminished for the second and third runs. This training effect was to be expected and was the reason why the scenarios were performed in a randomized order. In a study investigating the training effect of the d2, Bühner et al. [36] found an average increase of 13% when students repeated the d2 after approximately 35 min. A somewhat similar increase was found in our study. However, in the work by Bühner et al., students filled out a questionnaire, whereas our subjects performed physically intense high-quality CPR. Interestingly, there was no difference in the shifts of concentration performance between PPE groups. Given the randomization, this leads to the conclusion that none of the PPEs had a significate influence on the cognitive performance measured by the d2. Regarding the NHPT, we did not find such a training effect.

This study was conducted with experienced EMTs, who had approximately 3 months of experience of providing CPR in pandemic PPE at the time of the study. Results might differ in settings where the overall experience with CPR in PPE is different, and our findings should be extrapolated to such settings with caution.

Strengths of our trial include its triple-cross-over non-inferiority design, as well as the randomization of both the compilation of teams and the sequence of scenarios. Further studies are warranted to investigate the impact of various other kinds of PPE (such as CBRN equipment) on healthcare workers’ neurocognitive performance.

## Conclusion

Our findings indicate that concentration performance increases when performing BLS. In this regard, PPE including FFP masks is non-inferior to no masks. PPE should be used as indicated, without fear of impaired ability to perform complex medical tasks.

## Data Availability

The dataset used and/or analysed during the current study are available from the corresponding author on reasonable request.
